# A Special Chinese Reading Acceleration Training Paradigm: To Enhance the Reading Fluency and Comprehension of Chinese Children with Reading Disabilities

**DOI:** 10.3389/fpsyg.2016.01937

**Published:** 2016-12-12

**Authors:** Li Dai, Chenchen Zhang, Xiangping Liu

**Affiliations:** School of Psychology, Beijing Normal UniversityBeijing, China

**Keywords:** Chinese, reading acceleration training, morpheme, dyslexia, character, words

## Abstract

According to a number of studies, use of a Reading Acceleration Program as reading intervention training has been demonstrated to improve reading speed and comprehension level effectively in most languages and countries. The objective of the current study was to provide further evidence of the effectiveness of a Reading Acceleration Program for Chinese children with reading disabilities using a distinctive Chinese reading acceleration training paradigm. The reading acceleration training paradigm is divided into a non-accelerated reading paradigm, a Character-accelerated reading paradigm and a Words-accelerated reading paradigm. The results of training Chinese children with reading disabilities indicate that the acceleration reading paradigm applies to children with Chinese-reading disabilities. In addition, compared with other reading acceleration paradigms, Words-accelerated reading training is more effective in helping children with reading disabilities read at a high speed while maintaining superior comprehension levels.

## Introduction

The reading performance of children with a developmental reading disability (RD) is usually not smooth and not fully accurate (Compton and Carlisle, 1994; Horowitz-Kraus et al., 2014b). In past years, a number of studies have demonstrated that phonological processing defects are the primary reason for RD (Bradley and Bryant, 1983; Wagner and Torgesen, 1987; Snowling et al., 1991; Shaywitz et al., 1998; Ramus, 2003, 2006), and reading fluency is the consequence of effective phonological processing (Lyon and Moats, 1997). Phonological processing defects cause dyslexic children difficultly in extracting reading-decoding from grapheme to phoneme (Rack et al., 1992; Ackerman et al., 1994; Snellings et al., 2009). Word decoding is the prerequisite and foundation of reading comprehension in reading (Gough et al., 1996). Based on the above mentioned view, intervention investigating dyslexic children primarily focuses on how to catch them up in phonological processing (Snellings et al., 2009). However, data analysis of the training paradigm based on the phonological processing deficit has demonstrated that although the accuracy of word decoding is significantly improved, the intervention concerning reading fluency and reading comprehension is ineffective (Lyon and Moats, 1997; Torgesen et al., 2001; Wolf and Katzir-Cohen, 2001; Snellings et al., 2009). The current intervention regarding RD slightly changes direction; it not only emphasizes reading fluency and accuracy but is also utilized to maintain a certain reading comprehension level (Wolf, 2001; Karni et al., 2005).

Rapid automatic naming (RAN) training demonstrates that faster word reading makes dyslexic children improve the textual reading comprehension (Tan and Nicholson, 1997). In addition to the phonological processing deficit, the naming speed deficit is a cause of RD, and is described as slow single-word reading and naming (Korhonen, 1995; Wimmer et al., 1998; de Jong and van der Leij, 2003). RAN involves a series of complex cognitive processes, such as attention, perception, concept, phoneme, and muscle movement (Wolf and Bowers, 1999), by requiring children to name a series of items quickly and correctly in the short term. This approach is consistent with Perfetti (1985) verbal efficiency theory that interventions based on naming speed, which have been demonstrated to increase reading speed, can reduce the memory load of extracting letter pronunciation, causing the level of reading comprehension to improve (Snellings et al., 2009). In the process of reading intervention training, children read, at their own fastest speed, single letters and words that are stored in the psychological dictionary in the form of units, increasing their understanding of words and text under the theory of improving reading fluency (Horowitz-Kraus et al., 2014a). Among these methods, Breznitz (2006) first used a reading acceleration program to train reading speed to improve reading fluency while maintaining high comprehension.

The reading acceleration paradigm (RAP) refers to presenting letters or word on computer by a programmatic rate and has been shown to effectively improve word and text reading speed and reading comprehension in alphabet language systems. Following Breznitz’s (2006) accelerated training on Hebrew-speaking dyslexic adults, another training result as to Dutch dyslexic children and normal children showed that compared with non-accelerated training, RAP allowed children with RD to read at a high speed while maintaining a high comprehension level. Brain imaging evidence also indicates that the word rate in RAP can be used as an independent variable to influence reading comprehension and accuracy (Karni et al., 2005). At the same time, accelerated reading training may provide a supportive approach, stimulating the typical neural circuits associated with reading to increase the activation of the right frontal region and thereby compensate for a dyslexic child’s developing brain (Horowitz-Kraus et al., 2014b). A series of studies demonstrate that through the underlying mechanism of accelerated reading intervention, it is possible to improve the decoding processing word speed and reading comprehension for children with RD, regardless of the language (Korinth et al., 2009; Horowitz-Kraus and Breznitz, 2014; Horowitz-Kraus et al., 2014a). However, does the effectiveness of RAP in alphabet language training extend to Chinese children with RD?

The reading processing of Chinese characters is special and different from alphabetic languages due to the unique linguistic features of a character language (Wu et al., 2012); however, similar to children who speak alphabetic languages, Chinese children with RD are described as slow at word decoding and reading and inaccurate at spelling (Ho et al., 2002; Ho et al., 2004). The phonological processing deficit and naming speed deficit are two widely accepted cognitive deficits in both alphabet languages and Chinese (Denckla and Rudel, 1976; Shu et al., 2008), while the morpheme processing defect is a unique core deficit for Chinese children with RD (McBride-Chang et al., 2003; Shu et al., 2006; Tong et al., 2009). Using English and Chinese as comparative examples, reading in alphabetic language systems is based on the connection of phonemes and graphemic symbols, whereas reading in Chinese is base on the binding of meaningful morphemes and graphic units (Tan et al., 2000).

The morpheme, as the smallest binding unit in Chinese writing symbology, engages the perceptive ability of children for the smallest paired sound and meaning element and determines children’s semantic skills (Nagy et al., 2003). Most Chinese characters can comprise a compound word, which are themselves composed of different morpheme units; 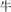/niu, for example, can be composed of :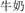/milk; 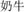/dairy; and 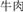/beef (McBride-Chang et al., 2003; Shu et al., 2006). Therefore, characters as basic writing units in Chinese (Tan et al., 2005) can be divided into separate precise meaning characters and vague sense characters. A character with a fuzzy meaning combined with other morphemes forms words with precise semantics (Tan et al., 2000). For example, the word “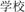/school” is a compound word consisting of two characters. One character is the morpheme “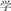/learning”, and another morpheme is the “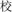/a place.” Even if children are not familiar with the word “
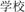
”, they can combine the semantics of the two morphemes to recognize the meaning of the word, “a place for learning.” Similarly, Tong et al. (2014) use the word “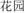/garden” as an example to show that the process of word reading in Chinese by forming the composition of the composite morpheme is different from other languages. It is estimated that more than seventy percent of Chinese words are compound words (Institute of Language Teaching and Research, 1986). In the current study, we divided Chinese reading into character reading and word reading for reading acceleration training of Chinese children with RD.

In the present study, to examine the effectiveness of the RAP for Chinese children with RD, we selected 51 dyslexic children and trained their reading speed and comprehension through use of the Chinese RAP. Considering the special features of the Chinese language, we used a control group and two types of the RAP: non-accelerated reading (a control group), character-accelerated reading and word-accelerated reading. We expect that the RAP is effective for Chinese children with RD and that the word-accelerated reading paradigm would be more effective for training Chinese children’s reading speed and comprehension.

## Materials and Methods

### Participants

This study concerns Chinese children with RD. The children were sampled from a total of 1036 students from two different primary education schools in Beijing. The rate of screening was 4.4%. The criteria applied to Chinese children with RD are as follows: according to the definition of dyslexia (Dryer et al., 1999), we used the Raven Test (Zhang and Wang, 1985) to exclude those children with a low IQ, and we used the Standardized Reading Comprehension Test (Wang et al., 2009) and the Amount of Primary Literacy Test (Wang and Tao, 1996) to define those students whose reading and literacy scores were 1.5 standard deviation lower than other students. We used the ADHD Conners Teacher Rating Scale (Yu et al., 2004) to exclude children with ADHD.

All participants were in Grades 3–4 in elementary schools with a mean age of 9 years, 4 months (*SD* = 1.08) and had no history of general learning, language learning, hearing, or vision problems. There was no indication of ADHD, and they had never repeated a grade. The majority of the participants have Chinese as their mother tongue and are right-handed. All participants were within the normal range of non-verbal IQ and passive vocabulary knowledge; the children with RD in the experimental and control groups were matched on these measures (**Table 1**). All participants and their guardians signed an informed consent form prior to participating in the experiment. The experiment was approved by the Academic Committee of the School of Psychology, Beijing Normal University, China.

**Table 1:** The homogeneity test of the experimental group and control group.

	The experimental group	Control group	*t*	*p*
Literacy amount	1976.143	1981.143	0.278	0.965
Reading comprehension	7.483	7	0.287	0.943
Raven’ score	40.58	40.73	0.273	0.943

### Design

All dyslexic children were told to participate in an accelerated reading training. The children were randomly divided into three training groups: a character-accelerated reading group (*n* = 17), a word-accelerated reading group (*n* = 17) and an un-accelerated reading group (*n* = 17). However, there were six students in un-accelerated reading group who were unwilling to continue to participate in further training after this first training. Therefore, we adjust the number of the student in each group. This left a character-accelerated reading group (*n* = 15), a word-accelerated reading group (*n* = 15) and an un-accelerated reading group (*n* = 15). The children were trained three times a week for 3 weeks with 32 sentences. Before and after training, a self-paced test and a fast-paced test to measure both reading speed and comprehension was administered.

#### Materials

Pre-tests, Post-tests and training materials were prepared from the Chinese teaching book. The level of the teaching materials that were selected to test and train was half a year higher than that of the children. There are 29 sentences in the Pre-and Post-tests along with multiple-choice reading comprehension questions; of those 29 sentences, five sentences were used for practice, and 24 sentences were used for formal experimentation. Because there are 32 different sentences used as stimulus materials in accelerated training, 358 sentences are required in each grade for a total of 716 sentences. Each sentence has 15 to 20 characters, a question and 4 possible answers. The context includes reasoning, memory, glossary, and summarization.

#### The Reading Acceleration Paradigm

The RAP is divided into three programs: non-accelerated reading, accelerated character reading and accelerated word reading. The difference between the three training groups is the speed of and the approach used for stimulus sentence presentation and the rate at which participants read. In each sequence, a “+” is first shown in the center of a black screen (500 ms) followed a blank screen (200 ms), and the stimulus sentences are subsequently presented.

In the non-accelerated reading paradigm, verbatim instructions are used to present the sentences. The children with RD were asked to start reading silently and immediately when the first character appears, and press the spacebar to advance at their own pace until the last character is read. In the accelerated character reading paradigm, the participants are automatically presented with completed sentences by computer consisting of several characters until the end of the last character in the sentence. The presentation time in the first training depends upon the average time in the practice phase; in the accelerated word reading paradigm, the participants are automatically presented with completed sentences consisting of several words by computer until the end of the last word in the sentence. Each word is composed of two to three single characters. The presentation time in the first training is decided by the average time elapsed in correctly answering comprehension questions in the practice phase. After the stimulus disappears, a blank screen is shown (200 ms), and the multiple choices (1, 2, 3, 4) corresponding to the sentence are subsequently given. The children with RD were asked to press the corresponding link to answer. Feedback for the correct answer was given with a red “√,” while feedback foe a wrong answer was given with a red “X.” The next sequence commences after a short break with a blank screen (200 ms). Each training program has 32 sentences. The decoding speed and the accuracy of reading comprehension is automatically assessed by the computer. In the two accelerated reading paradigms, if the children with RD can consecutively answer three multiple choice questions, the time during which characters are presented in the accelerated character reading paradigm will be reduced by 4 ms, and the time during which words are presented in the accelerated word reading paradigm will be reduced by 6 ms. Conversely, if the children with RD cannot consecutively answer three multiple choice questions, the time during which characters are presented in the accelerated character reading paradigm will be increased by 4 ms, and the time during which words are presented word in the accelerated word reading paradigm will be increased by 6 ms. The task was programmed and delivered using VB8.0 software. A 36 cm display device with a screen resolution of 1024 × 768 and a refresh rate of 85 Hz was used to present stimuli. Participants were placed approximately 60 cm from the screen.

### Pre-and Post-tests

A self-paced test and a fast-paced test, in the form of Pre-and Post-tests, were used to measure both reading speed and comprehension. In a self-paced test, as soon as the stimulus sentence appears on the screen, the children with RD start to read. When they have read the whole sentence, they are required to press a key on the keyboard to continue and choose from four options for answering a reading comprehension question. In a fast-paced test, after the sentence appears, the first character in the sentence begins to disappear from the left. The time of disappearance depends upon the average time that each character is read in practice phase. The contents of Pre-and Pro-tests are randomized. The purpose of the self-paced test and the fast-paced test is to measure the decoding speed and sentence comprehension level of the children with RD while engaged in self-paced reading and fast-paced reading.

## Results

We used a 2 × 3, two-way, mixed analysis of variance (ANOVA) with Training Group (un-accelerated reading group vs. character accelerated reading group vs. word accelerated group) as between-subjects factors and with Time (pre-test and pro-test) as a within-subjects factor (Descriptive statistics see **Table 2**). There was a significant main effect of Training Condition, *F*(2,42) = 29.69, *p* = 0.042, η = 0.47, indicating that accelerated training increased reading rate in both pre-test and pro-test. Finally, there was a significant main effect of Time, *F*(2,42) = 63.58, *p* = 0.001,η = 0.54, which was modified by a significant interaction effect between Time and Training Group with *F*(2,42) = 26.44, *p* = 0.006, η = 0.48.

**Table 2:** The reading speed and comprehension accuracy of pre-test and post-test in different accelerated reading training group.

Test		Non-acceleration (*n* = 15)		Character-acceleration (*n* = 15)		Words-acceleration (*n* = 15)	
		*M*	*SD*	*M*	*SD*	*M*	*SD*
Reading speed of a self-paced test	Pre-test	7736.5	1154.21	7116.21	2645.04	6245.69	2562.45
	Post-test	7615.54	2370.21	6127.15	3930.77	4093.54	1807.54
Reading accuracy of a self-paced test	Pre-test	63.67	12.61	63.2	11.77	72.92	8.13
	Post-test	65.52	17.62	77.41	7.5	75.76	8.17
Reading speed of a fast-paced test	Pre-test	5998.1	3215	5682.6	3419.5	3858.4	1469.9
	Post-test	5625.81	2252.88	4162.47	1158.29	3120.87	801.13
Reading accuracy of a fast-paced test	Pre-test	63.22	9.65	60.76	14.64	71.43	10.32
	Post-test	63.22	15.1	73.48	14.2	75.89	7.87

**FIGURE 1:**
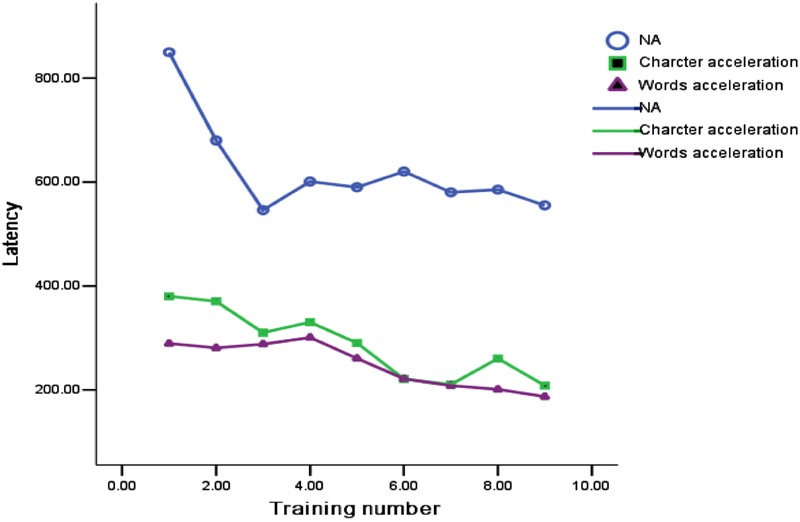
**The change of Chinese children with RD’s reading speed in the training process of three different accelerated reading groups**.

Further post-test analysis about the differences of the reading speed in three training groups found that the reading speed of un-accelerated reading group and accelerated character reading group have no significant difference in a self-paced test (*p* = 0.284) and a fast-paced test (*p* = 0.084). On the contrary, compared with un-accelerated reading group, the reading speed of the word accelerated reading group is significantly faster than the un-accelerated reading group children’s reading speed in a self-paced test (*p* = 0.015) and a fast-paced test (*p* = 0.005). In addition, to compare the children with RD’ reading speed during the different accelerated reading training, the **Figure 1** described the tendency of the reading speed of different training group. We found that at the first training, the accelerated character reading group and accelerated word reading group apparently use shorter time to read. It is until the fourth time that the reading speed is slowest, while after the fourth training, the trend presented accelerated reading until the end of the training. Non-accelerated reading training group has always behind the other accelerated reading group in the reading speed, although with the increase of the number, the reading speed get little increased. Most importantly, compared to un-accelerated group and the accelerated character reading group, the reading speed of the accelerated word reading group has remained at a high speed level.

**FIGURE 2:**
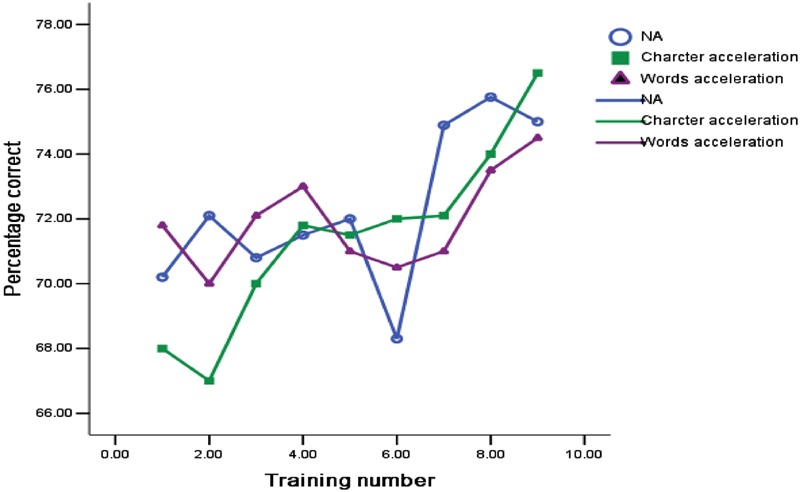
**The change of Chinese children with RD’s reading comprehension accuracy in the training process of three different accelerated reading groups**.

Further post-test analysis about the differences of the reading comprehension level in three training groups found that not only accelerated character reading training group (*p* = 0.01) but also accelerated word reading training group (*p* = 0.027) is significantly higher than the reading comprehension level of un-accelerated reading training group in self-paced test. However, there is no difference in accelerated character reading group and accelerated word reading group (all *p* > 0.05). In addition, to compare the children with RD’ reading comprehension level during the different accelerated reading training, the **Figure 2** described the tendency of the reading comprehension level of different training group. We found that the trend of un-accelerated reading training was less stable; the level of accelerated character reading group is lower than the level of accelerated word reading group at first to fourth time, and is consistent with the level of accelerated word reading group from the fifth. Most importantly, the level of reading comprehension always maintained at a more than 70 percent of the high level and stable tendency.

## Discussion

In the current study, we used the Chinese RAP (un-accelerated reading group, character accelerated reading group and word accelerated group) to train Chinese children with RD to examine its effectiveness and to explore whether accelerated character reading or accelerated word reading is a better fit for Chinese children with RD.

The results of our research first showed that in comparison with the non-accelerated reading group, the reading comprehension accuracy of the accelerated character reading group improved, while the reading speed and the reading comprehension level of the accelerated word reading group significantly increased. This finding may be the result of the difference between un-accelerated reading and accelerated reading – the reading time for children with RD in the un-accelerated reading group was under their manual control, while the reading time for children in the character accelerated and word accelerated reading groups was computer-controlled, and progressively shortened due to the increase in reading comprehension accuracy. Therefore, the children with RD in the accelerated reading groups were required to do their best to mobilize their cognitive resources as soon as possible such that they could complete the phonological and semantic processing connection from the graphic decode. In the self-paced test, there was no difference in reading speed between accelerated character reading and un-accelerated reading, while the accuracy in character accelerated reading was higher than in un-accelerated reading. This finding may be observed because in contrast to Chinese dyslexic children whose difficulties center on phonological awareness and naming speed, children with RD prefer visual processing and ignore reading speed. In accelerated character reading, each character is a single morpheme, and a writing unit is based on similarly defined graphics in the morpheme system (Tan et al., 2000); is why the level of reading comprehension in accelerated character reading is higher than in un-accelerated reading.

The study also demonstrated that in the normal reading mode, in comparison with the non-accelerated reading group, the accelerated word reading paradigm can significantly improve the reading speed and reading comprehension level of children with RD. This paradigm is consistent with the outcome of the training paradigm in alphabetical languages that computerized accelerated training can improve reading-decoding speed, reading fluency and accuracy while maintaining a high and stable level of reading comprehension (Karni et al., 2005; Korinth et al., 2009; Horowitz-Kraus and Breznitz, 2014; Horowitz-Kraus et al., 2014a). As a consequence, if the entire sentence is separated into several compound words to form precise semantics, it will have a beneficial effect on the reading fluency and level of reading comprehension of children with RD. Numerous studies demonstrate that it is difficult for Chinese children with RD to combine independent morphemes into meaningful compound words (e.g., Shu et al., 2006; McBride-Chang et al., 2011). The results of the current study may provide evidence that morpheme awareness is likely to be improved by the accelerated reading paradigm. In other words, the accelerated word reading paradigm requires children with RD to read the meaningful compound words rather than a single morpheme; to some extent, it will contribute to the automatic transformation of independent morphemes into precise and semantic words.

This study has several shortcomings. First, all participants are Chinese children with RD, meaning that additional training data on reading speed and comprehension may need to be collected in the near future. Second, due to subjective teaching in the Chinese educational system, the researchers have no way to perform a long intervention. In the near future, we may attempt to explore the effectiveness of the accelerated reading paradigm after months have passed. Meanwhile, we may increase the number and type of participants and the times of training and further confirm the effectiveness of the accelerated reading paradigm in those dyslexic children with various cognitive deficits.

## Author Contributions

CZ and XL designed the research; CZ and LD performed the experiments; DL analyzed the data; and LD and XL wrote the paper.

## Conflict of Interest Statement

The authors declare that the research was conducted in the absence of any commercial or financial relationships that could be construed as a potential conflict of interest.

The reviewer MD and the handling Editor declared their shared affiliation, and the handling Editor states that the process nevertheless met the standards of a fair and objective review.
